# Altered human voice processing in the frontal cortex and a developmental language delay in 3- to 5-year-old children with autism spectrum disorder

**DOI:** 10.1038/s41598-017-17058-x

**Published:** 2017-12-07

**Authors:** Yuko Yoshimura, Mitsuru Kikuchi, Norio Hayashi, Hirotoshi Hiraishi, Chiaki Hasegawa, Tetsuya Takahashi, Manabu Oi, Gerard B. Remijn, Takashi Ikeda, Daisuke N. Saito, Hirokazu Kumazaki, Yoshio Minabe

**Affiliations:** 1 Research Center for Child Mental Development, Kanazawa University, Kanazawa, 920-8640 Japan; 2 Gunma Prefectural College of Health Science, Maebashi, 371-0052 Japan; 3 Health Administration Center, University of Fukui, Fukui, 910-1193 Japan; 4 International Education Center, Kyushu University, Fukuoka, 815-8540 Japan

## Abstract

The inferior frontal and superior temporal areas in the left hemisphere are crucial for human language processing. In the present study, we investigated the magnetic mismatch field (MMF) evoked by voice stimuli in 3- to 5-year-old typically developing (TD) children and children with autism spectrum disorder (ASD) using child-customized magnetoencephalography (MEG). The children with ASD exhibited significantly decreased activation in the left superior temporal gyrus compared with the TD children for the MMF amplitude. If we classified the children with ASD according to the presence of a speech onset delay (ASD - SOD and ASD - NoSOD, respectively) and compared them with the TD children, both ASD groups exhibited decreased activation in the left superior temporal gyrus compared with the TD children. In contrast, the ASD - SOD group exhibited increased activity in the left frontal cortex (i.e., pars orbitalis) compared with the other groups. For all children with ASD, there was a significant negative correlation between the MMF amplitude in the left pars orbitalis and language performance. This investigation is the first to show a significant difference in two distinct MMF regions in ASD – SOD children compared with TD children.

## Introduction

Autism spectrum disorder (ASD) comprises a set of neurodevelopmental disorders characterized by deficits in social communication and restrictive and repetitive patterns of behaviour, interests, and activities. Language abilities are highly variable in individuals with ASD, with difficulties that range from mild to severe impairments in pragmatics and/or social communication^1^. Currently, language level is considered to be a continuous, rather than categorical variable^2^. Intriguingly, accumulating electrophysiological evidence suggests that deficits in the discrimination of rapid sound changes are associated with impaired speech processing in children with ASD^3^ as well as developmental language disorders^4–7^.

Auditory mismatch negativity (MMN) or its magnetic mismatch field (MMF)^8^ is quantified by subtracting the average waveform generated in response to standard stimuli from the average deviant waveform; MMN typically peaks between 100 to 250 ms from the onset of the stimulus change^9^. The sources of the MMN have been identified as the bilateral temporal regions in the primary and secondary auditory cortices, with contributions from the frontal regions^10–12^. In healthy populations, MMN/MMF is considered an indicator of change detection and has been used to probe speech discrimination^13–16^. Using source-localizing methods, MMN/MMF has been reported to be generated by temporal and frontal lobe sources, with the former being associated with change detection and the latter with involuntary switching of attention to sound changes^10^. Therefore, MMN/MMF have been used as neurophysiological markers for the integrity of auditory sensory memory and automatic change detection^6,17^.

In previous studies in children, the MMN latency ranged between 100 and 400 ms, depending on the age, cognitive skill and stimulus type^3,18–22^. MMN has been considered a suitable method to investigate speech development, even in infants, because MMN is elicited at all ages and is enhanced by native speech but not unfamiliar speech contrasts beginning at the age of 6 months^15,23^. Atypical MMN/MMF responses have also been reported in populations with developmental language disorders^4,24^ as well as children with ASD^3,22,25–37^.

Numerous auditory electrophysiological studies have primarily focused on MMF in older children with ASD (older than 6 years); however, to our knowledge, no previous magnetoencephalography (MEG) studies have focused on analysis of the MMF source in younger children with ASD (age 6 and under).

In the present study, we investigated the MMF evoked by voice changes during speech (i.e., a change in the fundamental frequency contour) in young children with ASD (aged 3–5 years) and a speech onset delay (AS-SOD), children with ASD without a speech onset delay (AS-NoSOD), and age-matched typically developing (TD) control participants. The aim of this study was to investigate regional activity in the brain during a speech perception task in order to explain the phenotypic heterogeneity in language development among children with ASD. Based on the finding that the atypical processing of auditory information is associated with language impairment in ASD, we hypothesized that young pre-school-aged children with ASD and a speech onset delay (SOD) exhibit atypical MMF compared with TD children and young children with ASD who do not have a SOD. We also hypothesized that atypical MMF would be associated with language performances at the time of recording, considering subjects’ ages and cognitive levels.

## Results

As shown in Table 1, forty-seven children with ASD and 46 TD children participated in the study.

**Table 1:** Demographic characteristics of the three groups.

	ASD Total	ASD-SOD	ASD-NoSOD	TD	One-way ANOVA (P-value)	Post hoc unpaired t-test (P < 0.05)
Number of participants	47	23	24	46		
Gender (male/female)	36/11	17/6	19/5	35/11		
Chronological age in months (range)	60.4 (40–72)	58.1 (40–72)	62.5 (40–72)	58.4 (37–79)	n.s.	
Head circumference (cm) (range)	51.1 (48.0–53.8)	51.0 (48.0–53.6)	51.1 (48.6–53.8)	51.0 (48.3–55.1)	n.s.	
**K-ABC**						
Mental Processing Scale (SD)	89.6 (20.4)	81.7 (21.3)	97.2 (16.6)	100.3 (10.6)	<0.05	ASD-SOD < ASD-NoSOD
						ASD-SOD < TD
Expressive Vocabulary (K-ABC) (SD)	15.2 (5.8)	12.3 (6.6)	18.0 (3.1)	16.7 (4.0)	<0.05	ASD-SOD < ASD-NoSODASD-SOD < TD
Receptive Vocabulary (PVT-R) (SD)	15.1 (9.7)	9.1 (6.5)	20.7 (8.9)	20.5 (9.8)	<0.05	ASD-SOD < ASD-NoSODASD-SOD < TD

K-ABC, Kaufman Assessment Battery for Children. The values indicate the mean values (range or standard deviation) for chronological age, head circumference, scales on the K-ABC, expressive vocabulary in the K-ABC and receptive vocabulary in the PVT-R. One-way ANOVA was used to determine the differences among the three groups (TD versus ASD-SOD versus ASD-NoSOD). n.s., not significant.

### Comparison of the MMF amplitudes in the 100–200 ms time window between the TD group and all ASD groups

**Figure 1:**
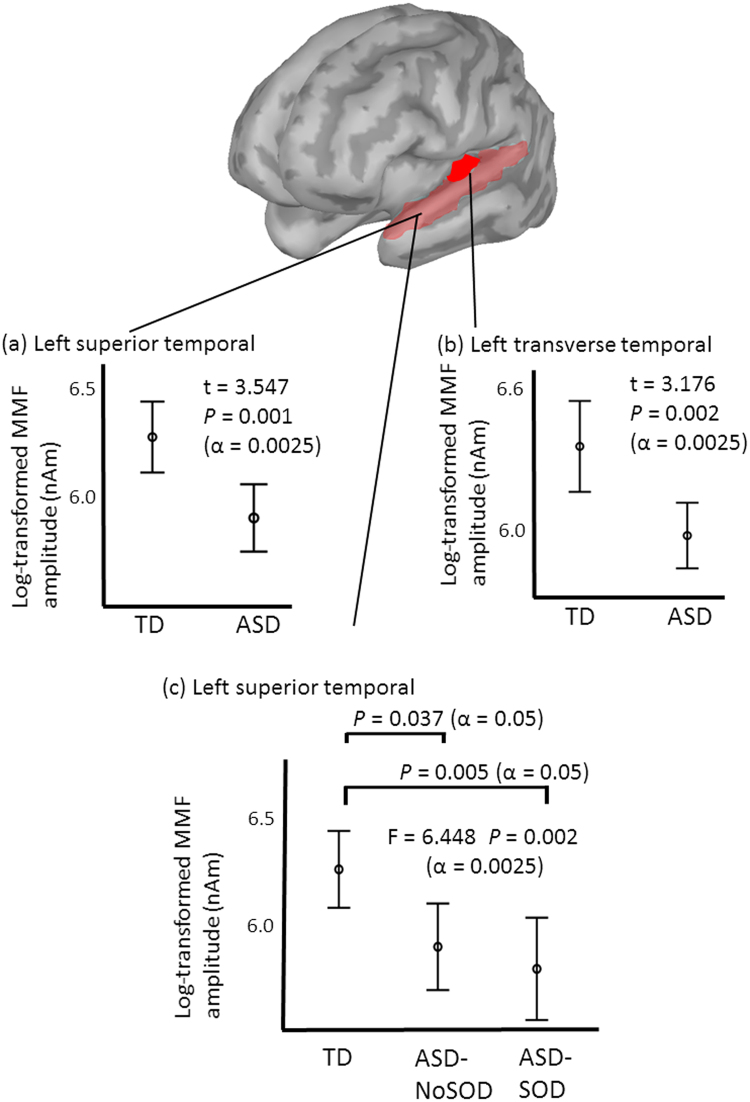
Comparison of the MMF source amplitude in the 100–200 ms time window. Significant differences were identified the left superior temporal gyrus (**a**) and left transverse temporal gyrus (**b**) between the TD children and children with ASD. There was a significant difference in the left superior temporal gyrus (**c**) between the TD children and ASD children with and without SOD. SOD, speech onset delay.

As shown in Fig. 1a,b, unpaired t- tests identified significant differences in the left superior temporal gyrus (t = 3.547, *P* = 0.001) and the left transverse temporal gyrus (t = 3.176, *P* = 0.002) in 20 regions of interest (ROIs) between the two groups (Bonferroni’s correction, alpha = 0.05/20 = 0.0025). As a complementary analysis (alpha = 0.05), the ANCOVA for these two ROIs, which included possible confounding factors (i.e., age^38^ and cognitive skill^39^) as covariances, identified significant differences in the left transverse temporal gyrus (F = 8.407, *P* = 0.005) and the left superior temporal gyrus (F = 11.227, *P* = 0.001) between the two groups. As another complementary analysis (alpha = 0.05) to test hemispheric lateralization, a repeated two-way ANOVA for these two ROIs, in which the group (i.e., TD and ASD) is between factor and the right/left (i.e., MMF from right or left hemisphere) is within factor, identified a significant interaction between these two factors in the left superior temporal gyrus (F = 4.415, *P* = 0.038) and left transverse temporal gyrus (F = 5.032, *P* = 0.027).

### Comparison of the MMF amplitudes in the 100–200 ms time window in the TD children and children with ASD with and without SOD

**Figure 2:**
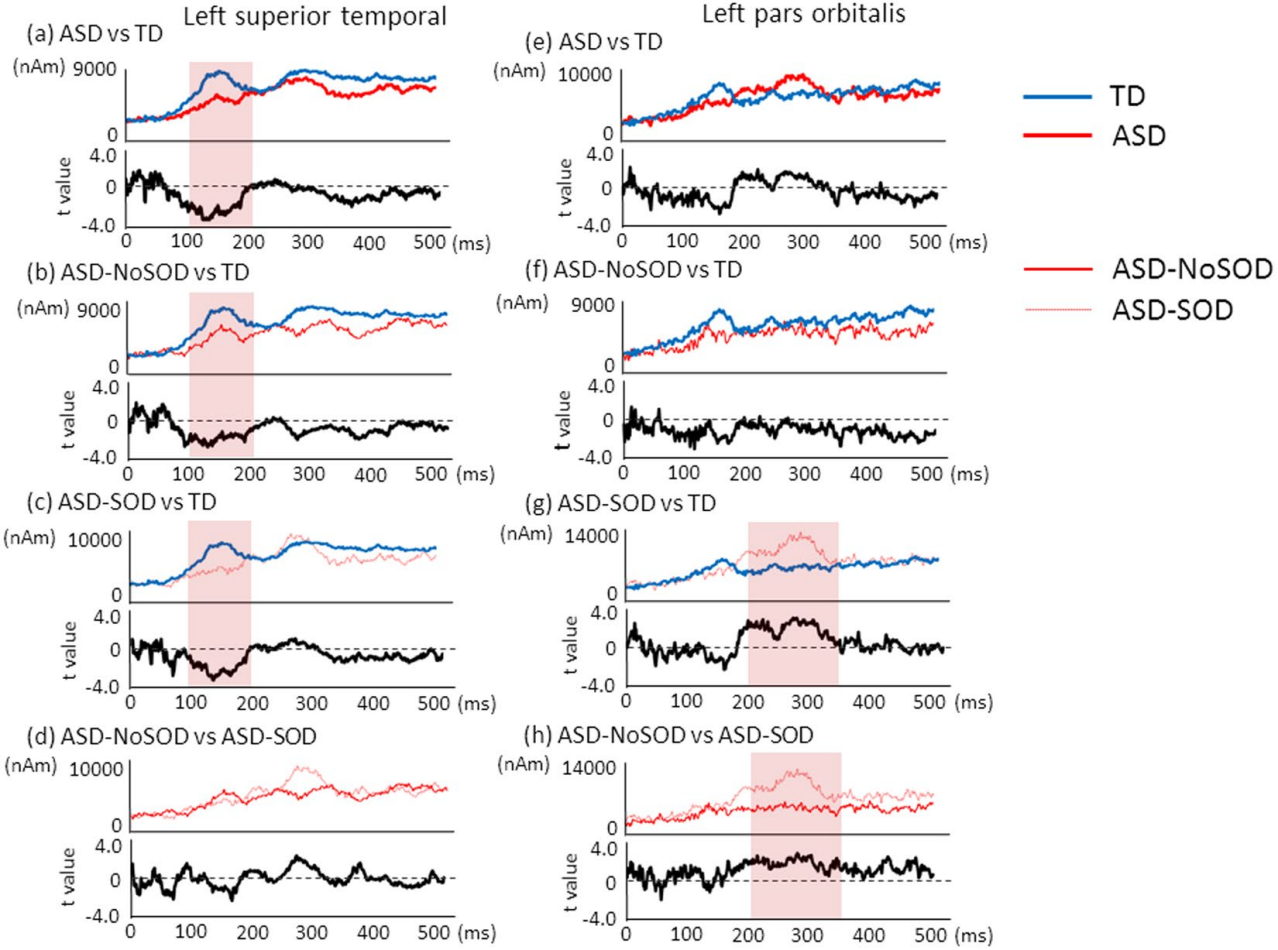
MMF source amplitude at each sampling point in the left superior temporal gyrus and left pars orbitalis. T values were calculated between the groups. Significant differences were indicated by a red coloured area. In the left superior temporal gyrus, (**a**) ASD vs TD, (**b**) ASD-NoSOD vs TD, (**c**) ASD-SOD vs TD and (**d**) ASD-NoSOD vs ASD-SOD. In the left pars orbitalis, (**e**) ASD vs TD, (**f**) ASD-NoSOD vs TD, (**g**) ASD-SOD vs TD and (**h**) ASD-NoSOD vs ASD-SOD. SOD, speech onset delay.

As shown in Fig. 1c, one-way ANOVA identified a significant difference in the left superior temporal gyrus between groups (F = 6.448, *P* = 0.002) (Bonferroni’s correction, alpha = 0.05/20 = 0.0025). A post hoc analysis (alpha = 0.05) identified significant differences between the TD children and the children with AS-NoSOD (*P* = 0.037), as well as between the TD children and the children with AS-SOD (*P* = 0.005). The ANCOVA (alpha = 0.05), which included age and cognitive skill as covariates, indicated a significant difference in the left superior temporal gyrus (F = 5.856, *P* = 0.004). The MMF amplitude and t-value for each sampling time point (1 ms) in the left superior temporal gyrus are presented in Fig. 2a–d.

### Comparison of the MMF amplitudes in the 200–350 ms time window between TD children and all ASD groups

Unpaired t-tests did not identify a significant difference in 20 ROIs in the 200–350 ms time window between the two groups (Bonferroni’s correction, alpha = 0.05/20 = 0.0025).

### Comparison of the MMF amplitudes in the 200–350 ms time window in TD children and children with ASD with and without SOD

**Figure 3:**
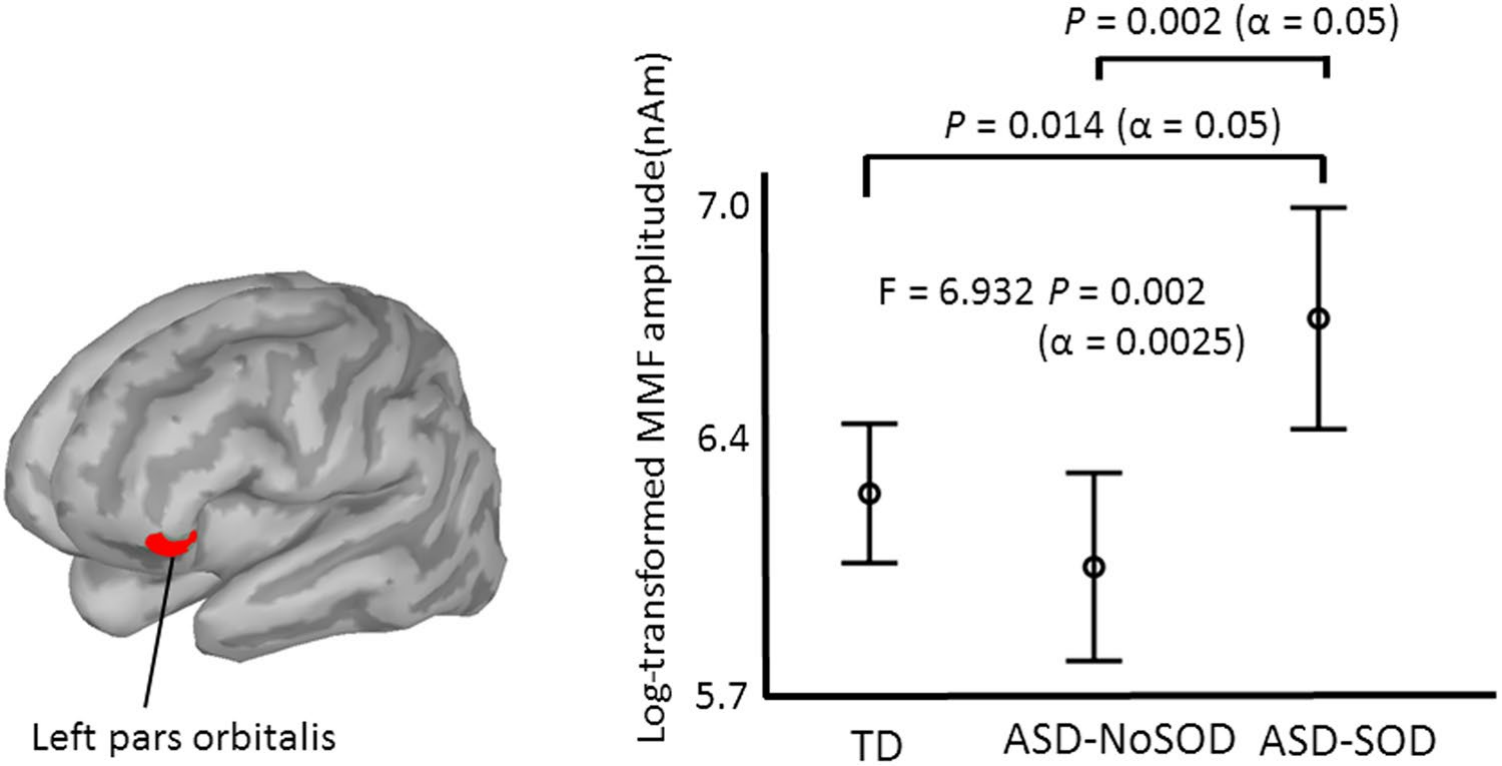
Comparison of the MMF amplitudes in the left pars orbitalis in the 200–350 ms time window among the three groups. One-way ANOVA identified significant differences among the three groups (F = 6.932 *P* = 0.002, α = 0.0025). The post hoc test identified significant differences between the TD children and children with ASD-SOD (*P* = 0.014, α = 0.05), as well as between the children with ASD-NoSOD and children with ASD-SOD (*P* = 0.002, α = 0.05).

As shown in Fig. 3, the one-way ANOVA revealed a main effect of the group on the left pars orbitalis (F = 6.932, *P* = 0.002) (Bonferroni’s correction, alpha = 0.05/20 = 0.0025). A post hoc analysis (alpha = 0.05) identified significant differences between the TD and ASD-SOD groups (*P* = 0.014), as well as between the AS-NoSOD and AS-SOD groups (*P* = 0.002). The ANCOVA (alpha = 0.05), which included age and cognitive skill, indicated a significant difference in the left pars orbitalis among the three groups (F = 6.957, *P* = 0.002). The MMF amplitude and t-value for each sampling time point (1 ms) in the left pars orbitalis are presented in Fig. 2e–h.

### Relationship between the MMF amplitudes and language performance

In the two regions with significant differences in the MMF amplitude among the three groups (i.e., the left superior temporal gyrus for the time window of 100–200 ms and left pars orbitalis for the time window of 200–350 ms), a Pearson’s correlation coefficient was used to investigate the relationship between the MMF amplitude and language performances (i.e., receptive and expressive vocabulary) for TD and ASD group, respectively. Bonferroni’s correction was applied for the two ROIs (i.e., alpha = 0.05/2 = 0.025).

In the TD children, Pearson’s correlation coefficient did not indicate a significant correlation between the MMF source amplitude in the left superior temporal gyrus and language performances (r = −0.287, *P* = 0.065 and r = −0.149, *P* = 0.323 for receptive and expressive vocabulary, respectively) or between the MMF source amplitude in the left pars orbitalis and language performance (r = −0.046, *P* = 0.770 and r = 0.267, *P* = 0.073 for receptive and expressive vocabulary, respectively).

**Figure 4:**
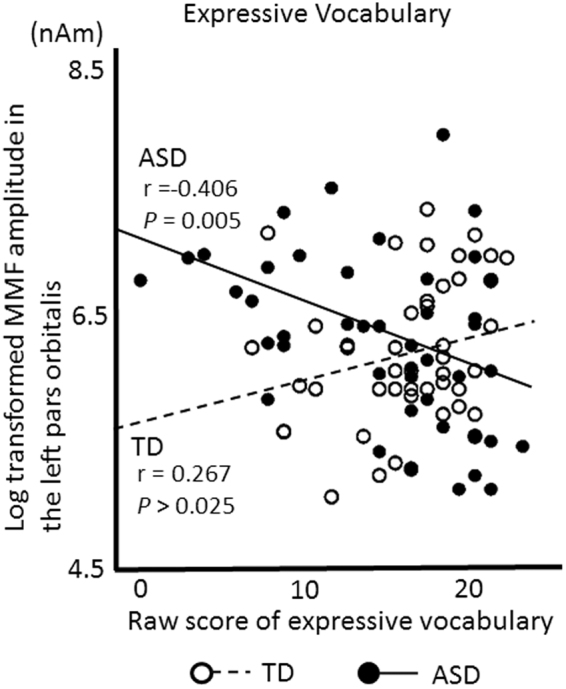
Relationship between the MMF amplitude in the left pars orbitalis and language performance. Scatter plots for expressive vocabulary. An open circle indicates the TD children and a closed circle indicates the children with ASD. The broken line indicates the regression line for the TD children and the bold line indicates the regression line for the children with ASD. Pearson’s correlation coefficient indicated a significantly negative correlation between the log-transformed MMF amplitude and the expressive vocabulary performance in the children with ASD (r = −0.406, P = 0.005, α = 0.025), but not in the TD children.

In children with ASD, Pearson’s correlation coefficient did not indicate a significant correlation between the MMF source amplitude in the left superior temporal gyrus and language performances (r = 0.110, *P* = 0.446 and r = 0.165, *P* = 0.266 for receptive and expressive vocabulary, respectively), however, there were significant correlations between the MMF amplitude in the left pars orbitalis and receptive vocabulary (r = −0.359, *P* = 0.014) and expressive vocabulary (r = −0.406, *P* = 0.005) (Fig. 4).

In addition, we employed a multiple linear regression analysis to predict the MMF amplitude (i.e., dependent variable) using group (i.e., TD and ASD), language performance (receptive or expressive vocabulary) and interaction term (i.e., group × language performance) as predictors to consider the potential interaction between group and language performance. We employed an alpha level of 0.05 for these complementary analysis using multiple linear regression analysis. As a result from expressive vocabulary ability, the multiple regression model suitably fitted the data (R = 0.364 F = 4.540, *P* = 0.005). Moreover, the interaction term (i.e., group × expressive vocabulary ability) showed significant in the left pars orbitalis (β = −0.337, *P* = 0.002). This result demonstrated that children with ASD and TD children have different relationships between MMF amplitude and expressive vocabulary ability. On the other hand, as a result from receptive vocabulary ability, the multiple regression model failed to fit the data (R = 0.291 F = 2.584, *P* > 0.05). Since there was a significant interaction between group and expressive vocabulary performance, we employed a stepwise multiple linear regression analysis to predict MMF amplitude (i.e., the dependent variable), using age and cognitive skill (first step) and expressive vocabulary (second step) as predictors (i.e., three independent variables) to consider the potential effects of age or cognitive skill on TD and ASD, respectively. We employed an alpha level of 0.05 for these complementary analyses using multiple linear regression. Expressive vocabulary was found to be significantly associated with MMF amplitude in the left pars orbitalis in the second step in children with ASD (Step 2, F = 3.031, β = −0.481, *P* = 0.012; Table 2). However, in TD children, there was no significant relationship between the MMF amplitude in the left pars orbitalis and any independent variable (i.e. expressive vocabulary, age, cognitive skills).

**Table 2:** Summary of the hierarchical regression analysis of the MMF amplitude in the left pars orbitalis according to the age, cognitive skills and expressive vocabulary of TD children and children with ASD.

	TD			ASD		
	β			β		
	Step 1	Step 2	t in Step 2	Step 1	Step 2	t in Step 2
Age (months)	0.031	−0.106	−0.617	−0.124	0.005	0.035
Cognitive skills	0.211	0.183	1.213	−0.162.	0.124	0.706
Expressive vocabulary		0.282	1.650		−0.481	−2.635**
Number of participants		45			46	
F	1.099	1.669		0.947	3.031*	
R	0.220	0.326		0.203	0.418	
Adjusted R^2^	0.004	0.043		−0.002	0.117	
ΔR^2^	—	0.058		—	0.134	

*P < 0.05 **P < 0.025.

## Discussion

The current study investigated the cortical pre-attentive discrimination of changes in speech tone in 3- to 5-year-old TD children and children with ASD. The amplitude of the early MMF (i.e., 100–200 ms) component was decreased in the left temporal auditory areas (i.e., the superior temporal and transverse temporal gyri) in the children with ASD compared with the TD children. This finding agrees with previous studies using electroencephalography (EEG) and MEG. In a previous EEG study of adult subjects, individuals with ASD did not exhibit a MMN response to emotional syllables and exhibited a reduced MMN to non-vocal sounds^40^. In addition, although our target age range (3–5 years) is missing from previous MMN/F studies, a large number of previous EEG/MEG studies have focused on MMN/F in older children with ASD^3,22,25,27–37,41,42^, of which many of the findings were highly inconsistent. Some studies reported larger MMN in children with ASD^22,29,33,36^, whereas other studies reported smaller^27,34,37^ or even normal MMN^35,42^ in children with ASD than in age-matched TD children. In part, these inconsistencies have been explained by differences in stimuli (e.g., vowel, tone, frequency change or intensity change)^22,29–31^, attentional condition (e.g., attended or unattended)^32^, time windows of focus (e.g., early MMN or late MMN including P3a)^22,30,33,35,36^ and age ranges^36^. The present study is the first to focus on younger children (3- to 5-year-olds) and replicated the decreased MMF amplitude reported in some previous studies in older children with ASD^27,34,37^ regardless of SOD. Moreover, this investigation is the first to show a reduction in the MMF amplitude in the left but not right hemisphere in children with ASD. This finding may be attributed to atypical brain lateralization in children with ASD, which was supported by recent MEG auditory-evoked field (AEF) studies in younger (3–7 year-old)^43^ and older (8–17 year-old)^44^ children with ASD.

In TD children, the Pearson’s correlation coefficient failed to demonstrate a significant correlation between the MMF source amplitude in the left superior temporal gyrus and receptive vocabulary ability (r = −0.287, *P* = 0.065), however, a higher MMF source amplitude tended to be associated with lower receptive vocabulary ability. Interpretation of this result is difficult because a deficit of MMN in children with specific language impairment was reported^45^. Further studies are necessary to conclude whether this finding could be replicated with a larger sample size in children with this age range.

If we divided the children with ASD into two groups according to the presence of SOD, the children with ASD-SOD exhibited enhanced cortical activation in the left inferior frontal gyrus (i.e., pars orbitalis) in the late MMF (i.e., 200–350 ms) compared with the two other groups. Consistent with our results, previous studies reported enhanced MMN or P3a (which is a late component of MMN and is thought to reflect an involuntary attention switch)^22,30,31,36^ in children with ASD. Vlaskamp *et al*. reported that children with ASD (age range: 8–12 year-old) exhibited a significant increase in P3a (latency: around 250 ms) in response to deviant stimuli compared with TD controls^30^, whereas another study reported a significant decrease in P3a in 7–11 year-old children (latency: around 340 ms)^22^. Intriguingly, Ferri *et al*. reported a higher P3a (latency: 220–240 ms) amplitude in ASD subjects during childhood (8 year-old), while the opposite was observed during young adulthood. These results, in conjunction with our findings, suggested that the enhanced late-component of MMF (time window: 200–350) in ASD subjects is a robust finding in younger children (3–5 year-old) with a delay in speech onset. A limitation of the present study is that it remains unclear whether the enhanced MMF in the frontal area is a signature of a developmental language impairment per se or mental retardation in the context of ASD; however, our findings from the multiple regression analysis (cognitive skill was employed as a confounding factor) suggest that the enhanced MMF observed in response to changes in speech tone is a neurophysiological feature of children with ASD and a developmental language impairment. Because auditory information processing in ASD is biased towards low-level information^46^, children with ASD and a language impairment may perceive the speech tone variations as low-level information and may fail to interpret the meaning it connotes. Therefore, these children have difficulties understanding variations in speech tone. Similarly, with respect to general speech comprehension, excellent pitch-perception skills may bias auditory processing towards perceptual, low-level information at the cost of processing speech at a higher level. One recent model claims that the excellent perception of low-level information at the cost of social skills in individuals with ASD^47^ may explain the enhanced frontal MMF activities (i.e., enhanced involuntary switching of attention to sound change) associated with lower language performance in children with ASD. Regarding the left inferior frontal cortex, interestingly, a previous fMRI study of school-age to adolescent subjects with ASD indicated that activation in the left inferior frontal gyrus increased during song stimulation compared with controls^48^. The left inferior frontal cortex may play an important role in the detection of rapid pitch changes in subjects with ASD at the cost of language performance.

There are several limitations of this study. First, we did not include subjects with a language impairment who did not present ASD symptoms; therefore, we could not conclude that the significant differences in the left temporal and frontal areas are specific neurophysiological markers for ASD-SOD. Second, we used only human voice stimuli; therefore, we cannot generalize our findings to other types of auditory stimulation. Third, there is a significant difference in current language abilities between ASD-SOD and ASD-NoSOD; therefore, the results from the regression analysis for current language abilities might be confounded by the history of speech onset delay. Fourth, we could not determine the precise location of the source of the magnetic field, but could have if we used individual anatomical images, such as those obtained from MRI. Future studies using child-friendly and open-type MRI devices to obtain individual fine brain structures are necessary to reduce the level of uncertainty in source level estimation. Despite these limitations, this investigation is the first to report differences in the brain source activity evoked by changes in speech tone between 3- to 5-year-old TD children and children with ASD with and without SOD.

## Materials and Methods

### Participants

The clinical group included 47 children who were recruited from Kanazawa University and prefectural hospitals in the Kanazawa or Toyama area. ASD was diagnosed according to the Diagnostic and Statistical Manual of Mental Disorders (4^th^ edition) (DSM-IV)^49^, the Diagnostic Interview for Social and Communication Disorders (DISCO)^50^, and the Autism Diagnostic Observational Schedule, Generic (ADOS-G)^51^; these assessments were conducted by a psychiatrist and a clinical speech therapist. Based on the criteria for SOD^52^, the children with ASD were divided into two groups based on the presence (AS-SOD) or absence of SOD (AS-NoSOD). Speech acquisition was considered typical if the parents reported that the child spoke his or her first single words before 24 months of age and his or her first two-word phrases before 33 months of age. Based on these criteria, 23 participants were defined as AS-SOD and 24 participants were defined as AS-NoSOD. The 46 TD children were matched to the ASD participants’ gender, age, and head circumference. No TD children had a history of SOD. Cognitive skills were assessed using the Japanese translation of the Kaufman Assessment Battery for Children (K-ABC)^53^. All participants were administered receptive and expressive language tests to investigate the relationship between the auditory brain response (i.e., the MMF) and language performance. Receptive vocabulary and comprehension were measured for each child using the Picture Vocabulary Test-Revised (PVT-R)^54^. The PVT-R is used to assess language comprehension and is similar to the Peabody Picture Vocabulary Test-Revised (PPVT-R). When an examiner speaks a word to a child in the PVT-R, the child chooses one of four pictures that represents the appropriate spoken word on each page. Expressive vocabulary was measured using the expressive vocabulary task in the K-ABC. In the expressive vocabulary task, the examiner shows the child several pictures, and the child provides the name of the objects. All participants had normal hearing according to their available medical records.

The parents agreed to allow their child to participate in the study and had full knowledge of the experimental nature of the research. Written informed consent was obtained prior to participation in the study. The Ethics Committee of Kanazawa University Hospital approved the methods and procedures, which were performed in accordance with the Declaration of Helsinki. The demographic data for all participants are presented in Table 1.

### Auditory-evoked field stimuli and procedures

**Figure 5:**
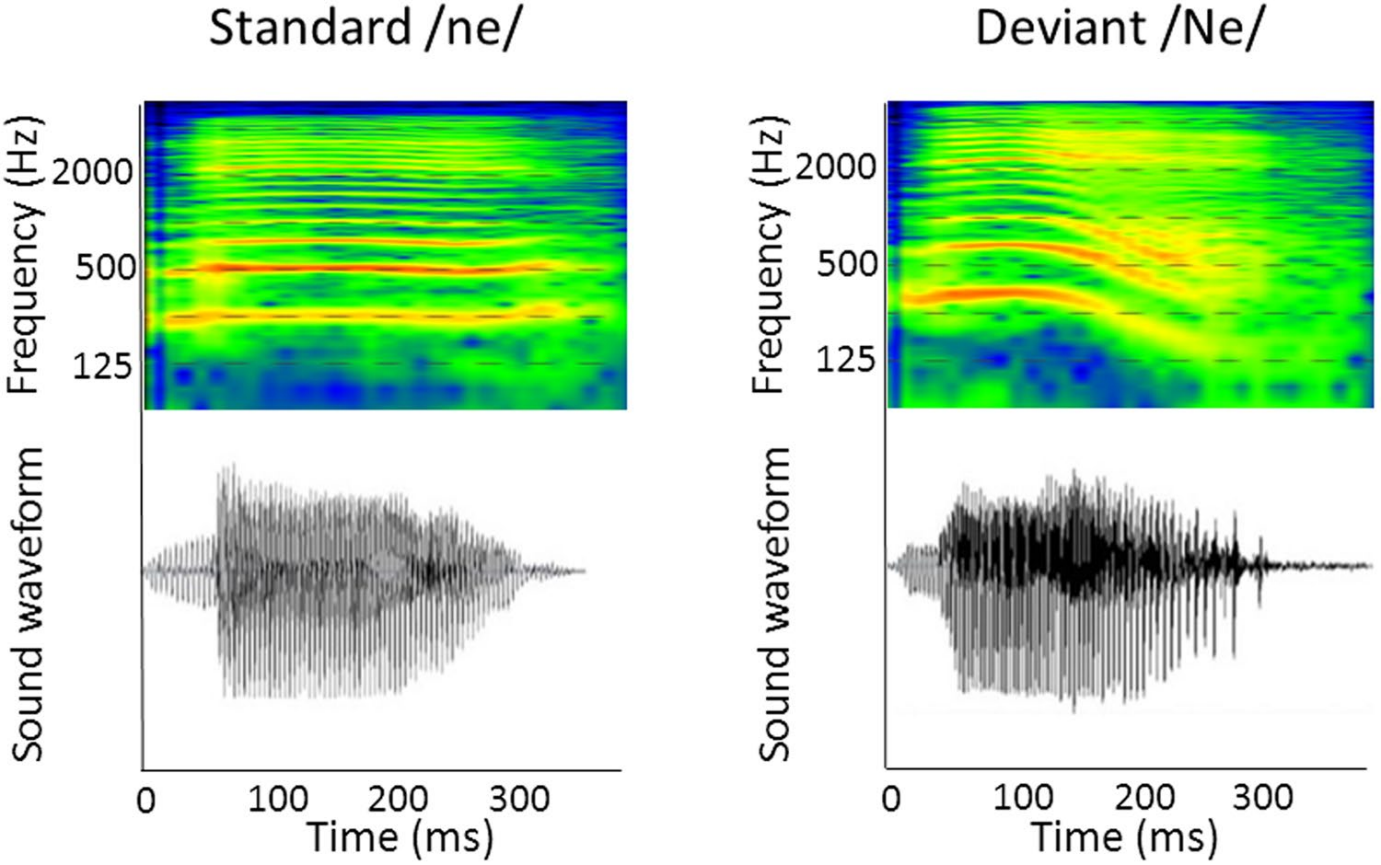
Spectral and temporal characteristics of the stimulus items *standard* /*ne*/ and *deviant* /*Ne/*. Formants are indicated with red lines. Please note the near-flat contour in the *standard* /*ne*/ (left) and the high falling contour in the *deviant* /*Ne*/ (right).

We used typical oddball sequences consisting of standard stimuli (456 times, 83%) and deviant stimuli (90 times, 17%). The stimuli consisted of the Japanese syllable “ne” pronounced two different ways (Fig. 5). A repetitive series of utterances of “ne” pronounced with a flat tone (/ne/) was used as the standard. This stimulus carries no intonational information. As a deviant stimulus, we used “ne” pronounced with a high falling tone (/Ne/), which carries intonational information (e.g., attention-seeking, emotional, declarative, or interrogative intonation). We used this syllable because /ne/ is a sentence-ending particle in Japanese and conveys prosodic information^55^. The syllable /ne/ is often used in mother-child conversations and expresses a speaker’s request for acknowledgement or empathy from the listener^56^. A female native Japanese speaker produced the /ne/ sounds, which were recorded using a condenser microphone (NT1-A; Rode, Silverwater, NSW, Australia) and a personal computer. The interstimulus interval (ISI) was 818 ms. Both stimuli had an intensity level of approximately 65 dB (A-weighted) at the head position against a background noise level of 43 dB. The intensity was measured using an integrating sound level metre (LY20; Yokogawa, Tokyo, Japan).

### Magnetoencephalography recordings

MEG data were recorded using a 151-channel SQUID (Superconducting Quantum Interference Device) whole-head coaxial gradiometer MEG system for children (PQ 1151 R; Yokogawa/KIT, Kanazawa, Japan) in a magnetically shielded room (Daido Steel, Nagoya, Japan) installed at the MEG Center of Ricoh Company, Ltd. (Kanazawa, Japan). One researcher remained in the room to encourage the children and prevent them from moving during the analysis. The location of the head relative to the helmet of the MEG device was measured using 3 coils attached on the head surface as fiducial points with respect to the landmarks (bilateral mastoid processes and nasion).

### Data analysis

The band pass-filtered MEG data (0.16–200 Hz) were collected at a sampling rate of 1000 Hz. The continuous MEG data were subsequently epoched into 100-ms pre-stimulus intervals and 900-ms post-stimulus intervals and baseline-corrected using the pre-stimulus interval. Epochs contaminated by muscle, heartbeat or eye blink artefacts that contained field amplitudes greater than ±4 pT were excluded from the analysis. Brainstorm^57^, which is documented and freely available for download online under the GNU general public license (http://neuroimage.usc.edu/brainstorm), was used for the subsequent analyses. Typical eye blinks and heartbeats were manually identified in the raw data for each participant to correct for blink and heartbeat artefacts. The pattern search function in Brainstorm was used to scan the raw data to identify other blinks and compute the average eye-blink topography across the MEG sensors. An eye-blink was modelled by the topography of its first PCA component. In addition to the heartbeat activity, the average heartbeat topography was also computed and modelled using the first PCA component. The trials of each type of stimuli were subsequently averaged after baseline correction (−50 to 0 ms). The mean averaging time for each stimulus (i.e., rare and frequent) was 76 ± 11 (mean ± standard deviation). The MMF responses were calculated by subtracting the average response to the standard stimuli (flat tone /ne/) from the average response to the deviant stimuli (falling tone /Ne/).

### Brain template

We could not obtain individual brain structural data because it is difficult to perform MRI recordings on young children without sedation. Instead, we estimated the brain structures based on the individual head surface shapes of the participants using a modified version of the estimation algorithm developed in our previous study^58^ and superimposed the coordinate system of the MEG on the resulting anatomical information. Our algorithm was developed to identify an optimal structural image from 98 brain examples using head surface points^59^.

### Analysis of the MMF source

We estimated the signal source of the MMF using the individually estimated anatomies of the children. The anatomical locations of the activating regions were based on the Desikan-Killiany gyrus atlas provided by FreeSurfer (open-source software: http://surfer.nmr.mgh.harvard.edu/)^60^. Source reconstruction was performed with Brainstorm^57^. To estimate the brain sources, we used an anatomically constrained MEG approach that places an anatomical constraint on the estimated sources by assuming that the recorded brain activity of each individual lies in the cortical mantle^61^. The lead field was then computed using the overlapping spheres algorithm^62^, with a cortical surface tessellated with 15,000 vertices. The inverse solution was calculated for each individual using Tikhonov-regularized minimum-norm estimates (MNE)^63^. A noise covariance matrix was calculated from the MEG recordings obtained in the −50 to 0 ms time window.

### Selection of regions of interest

The MNE source maps were obtained for each participant and group and averaged onto the cortical regions corresponding to the Desikan-Killiany gyrus atlas. The definition of the anatomical ROIs was based on the prediction that MMF generators would be located primarily in the temporal, frontal and parietal regions^9,64–68^. Twenty ROIs of the total of 68 ROIs of Desikan-Killiany gyrus atlas were selected for further analysis. Two temporal windows (100–200 ms and 200–350 ms) were selected in each ROI.

### Statistical analysis

Statistical analyses were conducted using SPSS (Statistical Package for the Social Sciences) for Windows, version 20.0 (IBM, Tokyo, Japan). For the 20 ROIs defined in the two time windows, differences in the log-transformed MMF amplitude were tested using unpaired t-tests (i.e., TD versus ASD) and one-way analysis of variance (ANOVA) (i.e., TD versus AS-SOD versus AS-NoSOD). Based on the number of ROIs, which were predefined according to previous studies^9,64–68^, Bonferroni’s correction was applied for the alpha level (i.e., alpha = 0.05/20 = 0.0025). If there was a significant difference in the MMF component amplitude among groups, for ANOVA (i.e., TD versus AS-SOD versus AS-NoSOD) result, we used post-hoc t test (alpha = 0.05). In addition, we included an analysis of covariance (i.e., ANCOVA) that included clinical variables (i.e., age and cognitive skills assessed by the K-ABC) as covariates to consider the potential confounding factors, such as an aging effect, on the AEF component in young children^69^. We employed an alpha level of 0.05 for this complementary analysis. We also used multiple linear regression analysis to predict MMF amplitude (i.e., the dependent variable) using group (i.e., TD and ASD), language performance (receptive or expressive vocabulary) and interaction term (i.e., group × language performance) as predictors to consider the potential interaction between group and language performance. We employed an alpha level of 0.05 for these multiple linear regression analyses. If a significant interaction between group and language performance was found, we employed stepwise multiple linear regression analysis to predict MMF amplitude (i.e., the dependent variable), using age and cognitive skill (first step) and language performance (i.e., receptive or expressive vocabulary) (second step) as predictors (i.e., three independent variables) for TD and ASD, respectively. We employed an alpha level of 0.05 for this complementary analysis.

### Data Availability

The datasets used and analyzed during current study are available from the corresponding author on reasonable request.
